# Association of Anthropometric Indices With Metabolic Phenotypes of Obesity in Children and Adolescents: The CASPIAN-V Study

**DOI:** 10.3389/fendo.2019.00786

**Published:** 2019-12-03

**Authors:** Moloud Payab, Mostafa Qorbani, Nazila Shahbal, Mohammad Esmaeil Motlagh, Shirin Hasani-Ranjbar, Hoda Zahedi, Gita Shafiee, Hasan Ziaodini, Mohammad Ali Pourmirzaiee, Ramin Heshmat, Roya Kelishadi

**Affiliations:** ^1^Obesity and Eating Habits Research Center, Endocrinology and Metabolism Molecular-Cellular Sciences Institute, Tehran University of Medical Sciences, Tehran, Iran; ^2^Non-communicable Diseases Research Center, Alborz University of Medical Sciences, Karaj, Iran; ^3^Endocrinology and Metabolism Research Center, Endocrinology and Metabolism Clinical Sciences Institute, Tehran University of Medical Sciences, Tehran, Iran; ^4^Non-Communicable Diseases Research Center, Endocrinology and Metabolism Population Sciences Institute, Tehran University of Medical Sciences, Tehran, Iran; ^5^Department of Pediatrics, Ahvaz Jundishapur University of Medical Sciences, Ahvaz, Iran; ^6^Department of Clinical Nutrition, School of Nutritional Sciences and Dietetics, Tehran University of Medical Sciences, Tehran, Iran; ^7^Chronic Diseases Research Center, Endocrinology and Metabolism Population Sciences Institute, Tehran University of Medical Sciences, Tehran, Iran; ^8^Health Psychology Research Center, Education Ministry, Tehran, Iran; ^9^Department of Pediatrics, Child Growth and Development Research Center, Research Institute for Primordial Prevention of Non-communicable Disease, Isfahan University of Medical Sciences, Isfahan, Iran

**Keywords:** anthropometric indices, abdominal obesity, general obesity, metabolic syndrome, obesity

## Abstract

**Background:** Obesity, particularly in the upper part of the body, is a major health problem. Measuring the neck circumference (NC) and wrist circumference (WrC) is a relatively new method of differentiating between normal and abnormal fat distributions. This study aimed to evaluate the association of NC, hip circumference (HC), and WrC with different phenotypes of obesity and their metabolic status.

**Methods:** In this multi-centric cross-sectional study, 4,200 students aged 7–18 years were selected from 30 provinces in Iran in 2014 by using a multistage cluster random sampling method. Metabolic syndrome (MetS) was defined based on the ATP III criteria modified for the pediatric age group. The subjects were classified into four groups according to their weight and metabolic status: metabolically healthy obese (MHO), metabolically non-healthy non-obese (MNHNO), metabolically non-healthy obese (MNHO), and metabolically healthy non-obese (MHNO).

**Results:** Significant but different associations of NC, HC, and WrC with obesity phenotypes were documented in the entire population. Significant but different associations of NC, HC, and WrC with metabolic phenotypes were also found in the entire population. In the multinomial logistic regression, the association of the different obesity phenotypes with the study anthropometric indices increased significantly with increasing NC, WrC, and HC. Also, per one unit increment in NC, HC, and WrC, the odds of MHO, MNHNO, and MNHO increased compared to that of the MHNO phenotype.

**Conclusion:** In children and adolescents, HC, NC, and WrC are significantly associated with obesity phenotypes and their metabolic status, and these metrics are suggested to be innovative, low-cost, and alternative tools for assessing them in different age and sex pediatric age groups.

## Introduction

The prevalence of obesity is increasing to pandemic proportions in the world. In 2014, the WHO reported that 52% of adults worldwide are overweight and obese (1). Also, childhood obesity has become a public health priority worldwide. The prevalence of overweight and obesity is also rising among children and adolescents in developing countries, rising from 8.1 to 12.9% in boys and from 8.4 to 13.4% in girls in 2013 (2). Also, approximately one-fourth of children worldwide are obese or overweight (3).

The pathophysiology of obesity is complex and involves the interaction of various genetic, metabolic, environmental, and behavioral factors (4). Among these factors, environmental factors are the most common cause of the epidemic of obesity, for example, a sedentary lifestyle (computer games and watching TV) and nutritional disorders (consumption of high-calorie foods and inappropriate diets) (5, 6).

In an article published in 2017, the World Federation of Obesity claimed that “early diagnosis and treatment of childhood obesity should be regarded as similar to vaccination” (7).

Along with weight gain, fat accumulation also increases in the body and causes various illnesses (8). Similarly, the majority of obese people have type 2 diabetes, insulin resistance, high blood pressure, and dyslipidemia. However, about 10–25% of obese people are metabolically healthy. These subjects with excess body fat are free of metabolic abnormalities. Hence, people who are metabolically “unhealthy” should be distinguished from those with the “healthy” obese phenotype (9–12).

Different anthropometric indicators are used to determine general obesity and abdominal obesity.

Body mass index (BMI) is a common anthropometric indicator for assessing weight status in adults and children (6, 13). This indicator has advantages, including the ease of measuring and interpreting it; however, this indicator also has limitations. It cannot differentiate the fat mass (FM) from the free fat mass (FFM), cannot indicate body fat distribution, and requires some calculations, and excess FM may conceal FFM deficits (6, 14).

Hip circumference (HC) reflects the body composition to a certain extent (i.e., muscle mass and fat mass), but in childhood, its prognostic value for later health risks in adulthood is limited (15). Several studies have shown that some other anthropometric indices, including waist circumference (WC), waist-to-hip ratio (WHR), and waist-to-height ratio (WHtR), are useful for determining abdominal obesity (14). The use of WHtR for detecting central obesity and the health risks associated with it was first proposed in the mid-1990s (16). The WC has been criticized for not taking into account differences in body height, and the WHtR is a better predictor of cardiovascular risk, visceral fat, and mortality (17). It is important to note that there is a progressive increase in WHtR with increasing body fat so that the value of the WHtR alone will not work as a predictor of body fat. WHtR is a rapid and effective indicator of health risks of obesity. WHtR is more sensitive than BMI as an early warning of health risks and is easier and cheaper to measure and calculate than BMI (18).

The use of these indices has certain limitations. The first measurement should be without clothes. Secondly, their values may vary along with fasting or satiety during the day, and it is difficult to measure these indices and requires calculation (19). Also, measuring these anthropometric indices is not easy in large studies, and there may be errors in measuring two parts of the body and calculating their ratio. WHR is generally considered a good tool to distinguish between different types of fat distribution, as it is highly correlated with visceral fat and plasma lipid concentrations. One of the disadvantages of WHR is that a reduction in weight usually results in a reduction in both WC and HC, so that WHR may not decrease despite the leaner body composition. In addition, a decrease in WHR may not be related to a reduction in cardiovascular risk factors. Finally, the decrease of WHR with age, especially in girls, is due to an increase in pelvic diameter and predominant fat deposition in the gluteal area. Therefore, other indicators such as wrist circumference (WrC) and neck circumference (NC) are considered (20–22).

Nowadays, NC is considered an indicator of the distribution of subcutaneous fat in the body. This indicator is a simple, fast, and easy anthropometric measurement that saves measurement time in population-based studies (23). One of the benefits of measuring NC is that it does not change during the day (4). Also, this indicator is not affected by respiration (breathing) or stomach fullness (24). Some studies have shown that an increase in NC is associated with metabolic disorders (1).

The WrC is another simple anthropometric indicator that is easily measured and can show bone size without being severely confounded by other tissues. WrC is easy to detect without the need for calculation and without disturbing the clothing during measurement, and some studies have also shown a significant association between WrC and metabolic disorders. However, this association should be investigated in different age groups (25, 26).

The aim of this study was to determine the association of NC, HC, and WrC with obesity phenotypes and their metabolic status.

## Methods

This study is part of the CASPIAN-V study conducted in 30 provinces of Iran in 2015; the details of the study protocol have been described previously (27). This study was conducted on 14,400 students at national level, from which 4,200 students were randomly selected for biochemical tests. The samples included students aged 7–18 years in primary and secondary schools in urban and rural areas throughout the country.

Subsequent to the selection of the qualified students, the aim of the study was explained to them. Also, their parents were invited to take a role after the study selection. Two sets of questionnaires were completed for students and their parents. The students' questionnaire was obtained from the World Health Organization–Global School Student Health Survey (WHO-GSHS) and was translated into Persian. The validity and reliability of the questionnaire were previously assessed (28, 29).

Weight was measured with minimal clothing, with 0.1 kg accuracy on a SECA digital weighing scale (SECA, Germany), and standing height was recorded without shoes, with 0.1 cm accuracy (SECA, Germany). Body mass index (BMI) was calculated by dividing weight (kg) by height squared (m^2^). WHO growth charts were used to categorize BMI. Waist circumference (WC) was measured using a non-elastic tape on the distance around the mid-point between the lower margin of the last palpable rib and the top of the iliac crest at the end of normal expiration, to the nearest 0.1 cm. The widest part of the buttocks was measured to obtain HC to the nearest 0.1 cm. WrC was measured by placing the superior border of the tape measure distal to the prominences of radial and ulnar bones, with 0.1 cm accuracy. NC was measured with the most prominent portion of the thyroid cartilage taken as a landmark, with 0.1 cm accuracy. Waist-to-height ratio was calculated as WC (cm) divided by height (cm) (29, 30).

Blood pressure (BP) was measured using a mercury sphygmomanometer on the right arm in a sitting position. It was measured twice at 5-min intervals, and the average was reported (31). High blood pressure is classed as a high measure in either systolic or diastolic pressure or both. The weekly frequency of physical activity (PA) was measured as the number of days on which the children reported that activity for at least 30 min outside of school had caused heavy sweating or large increases in breathing or heart rate. For the sake of statistical analysis, each weekly frequency received a classification [ <2 days per week (mild), 2–4 days per week (moderate), and >4 days (severe)].

To assess screen time (ST) behaviors, the students were asked to report how many hours per day they spent watching television, videos, or both, using their personal computer or playing electronic games. The total cumulative time spent for ST was calculated accordingly.

After a 12-h overnight fast, 6 ml of venous blood was collected and stored at −70°C. Fasting blood glucose (FBG), triglyceride (TG), total cholesterol (TC), low-density lipoprotein cholesterol (LDL-C), and high-density lipoprotein cholesterol (HDL-C) were measured enzymatically by Hitachi auto-analyzer (Tokyo, Japan).

In this study, we used the WHO growth curves to define BMI categories, i.e., an age- and sex-specific BMI in the 5th−85th percentile was considered normal weight, and a ≥95th percentile age- and sex-specific BMI was considered general obesity. Abdominal obesity was defined as waist-to-height ratio ≥0.5.

The subjects were classified into six groups according to general and abdominal obesity:
normal (5th < BMI < 85th percentile and WHtR < 0.5),
abdominal obesity (WHtR>0.5),
general obesity (BMI > 95th),
only abdominal obesity (WHtR > 0.5 and BMI < 95th percentile),
only general obesity (BMI > 95th and WHtR < 0.5),
and combined obesity (BMI > 95th and WHtR > 0.5).

The participants were classified as having metabolic syndrome (MetS) if they had three or more out of five criteria according to the Adult Treatment Panel III (ATP III) criteria modified for the pediatric age group (32, 33).

1. Abdominal obesity, as defined as waist-to-height ratio ≥ 0.5 (34).
2. FBG ≥ 100 mg/dL
3. Serum TG ≥ 100 mg/dL
4. HDL-C ≤ 40 mg/dL
5. BP > 90th age, sex and height specific percentile.

The participants were classified into four groups according to their weight and metabolic status:

Metabolically healthy obese (MHO): defined as “those not having metabolic syndrome (i.e., no more than two risk factors) and obese” (35).

Metabolically non-healthy non-obese (MNHNO): “non-obese and with metabolic syndrome).”

Metabolically non-healthy obese (MNHO): “both obese and with metabolic syndrome.”

Metabolically healthy non-obese (MHNO): “not having metabolic syndrome (i.e., no more than two risk factors) and non-obese)” (35).

### Statistical Analysis

Qualitative variables were reported as numbers and percentages, and quantitative variables were reported as mean ± SD (standard deviation). The Chi-square test was used to analyze qualitative variables, and comparison of means of quantitative variables was performed by Student's *t*-test.

Crude and age–sex-adjusted mean values of NC, HC, and WrC across different phenotypes of obesity were assessed using analysis of variance (ANOVA) and analysis of covariance (ANCOVA), respectively.

Multinomial logistic regression analysis was performed to evaluate the association of HC, NC, and WrC with different phenotypes of obesity and metabolic syndrome. Two models were defined: Model I represented the crude association (without adjustment), and Model II was after adjustment for age, sex, PA, ST, socioeconomic status (SES), and living area.

Data were analyzed using the STATA package version 11.0 (Stata Statistical Software: Release 11. StataCorp LP. Package, College Station, TX, USA), and *P* < 0.05 was considered as statistically significant.

## Results

In this study, 14,274 students (50.6% boys, 49.4% girls) and one parent of each performed the survey (out of 14,440). The participation rate was 91.5% for taking blood samples from students (3,843 out of 4,200 students selected for blood sampling). The mean ages of boys and girls were 12.4 (SD: 3.1) and 12.2 (SD: 3.2) years, respectively. 71.4% of students were from urban and 28.6% were from rural areas. Most students had low PA levels (58.2% of all students), which was different between boys and girls (56.2% boys and 60.4% girls) (*P* < 0.001) and also between urban and rural areas (58.8% in urban areas and 56.7% in rural areas) (*P* < 0.05). The prevalences of overweight (BMI > 85th), general obesity (BMI > 95th), and abdominal obesity (WHtR > 0.5) were 9.4, 11.4, and 21.1%, respectively. The prevalence of metabolic syndrome was 5%. Also, the prevalence of overweight in metabolic syndrome subjects was 8.3% (91.7% healthy subjects). The prevalences of only general (BMI > 95th and WHtR <0.5) and only abdominal obesity (WHtR > 0.5 and BMI <95th percentile) were 2.5 and 12.2%, respectively. Also, 9.8% of students had both general and abdominal obesity; the prevalences of MHO and MNHO were, respectively, 9.1 and 1.8%.

The characteristics of the participants, including their location of residence (urban-rural), physical activity, screen time, and socioeconomic status, according to age and sex are presented in Table 1. Physical activity levels were significantly higher in urban areas than in rural areas and were higher in boys than in girls (*P* < 0.001).

**Table 1:** Characteristics of participants according to sex and age: The CASPIAN-V study.

**Variables**		**Total** ***n*** **(%)**			**<10 years** ***n*** **(%)**			**≥10 years** ***n*** **(%)**		
		**Boys**	**Girls**	***P*-value**	**Boys**	**Girls**	***P*-value**	**Boys**	**Girls**	***P*-value**
Region	Urban	5,150 (71.3)	5,044 (71.6)	0.67	1,102 (69.5)	1,221 (67.1)	0.068	4,048 (71.7)	3,823 (73.2)	0.051
	Rural	2,078 (28.7)	2,002 (28.4)		483 (30.5)	599 (32.9)		1,595 (28.3)	1,403 (26.8)	
PA^a^	High	3,138 (43.8)	2,768 (39.6)	<0.001	738 (47)	706 (39.1)	<0.001	2,400 (43)	2,062 (39.8)	0.001
	Low	4,020 (56.2)	4,215 (60.4)		833 (53.1)	1,098 (60.9)		3,187 (57)	3,117 (60.2)	
ST^b^	High	5,863 (83.4)	5,781 (84.3)	0.077	1,310 (85.2)	1,531 (86.1)	0.250	4,553 (82.9)	4,250 (83.6)	0.175
	Low	1,164 (16.6)	1,079 (15.7)		228 (14.8)	248 (13.9)		936 (17.1)	831 (16.4)	
SES^c^	Low	2,325 (33.6)	2,234 (33.3)	0.094	518 (33.9)	544 (31.5)	0.032	1,807 (33.5)	1,690 (34)	0.404
	Moderate	2,343 (33.8)	2,172 (32.4)		509 (33.3)	543 (31.4)		1,834 (34)	1,629 (32.8)	
	High	2255 (32.6)	2297 (34.3)		500 (32.7)	642 (37.1)		1755 (32.5)	1655 (33.3)	

a *PA, physical activity*.

b *ST, screening time*.

c *SES, socioeconomic status*.

Table 2 shows the mean (95% CI) of HC, NC, and WrC according to general and abdominal obesity. According to this table, significant but different associations were observed of NC and HC with general and abdominal obesity in the entire population. The average values of NC, HC, and WrC were significantly higher among subjects with both abdominal obesity and general obesity. Also, in subjects with only general obesity, the average values of NC, HC, and WrC were higher than in those with only abdominal obesity.

**Table 2:** Mean (95% CI) of hip, neck, and wrist circumference according to obesity phenotypes: the CASPIAN-V study.

		**Normal^**a**^ (*n* = 10,783)**	**Only abdominal obesity^**b**^ (*n* = 1,716)**	**Only general Obesity^**c**^ (*n* = 355)**	**Combined obesity^**d**^ (*n* = 1,250)**	***P*-values between groups**	***P*-values within groups (*post-hoc*)**	**Abdominal obesity (*****n*** **=** **2,972)**^**e**^		**General obesity (*****n*** **=** **1,615)**^**f**^	
								**Yes**	**No**	**Yes**	**No**
Neck circumference (cm)	Crude	29.26 (29.19–29.33)	30.59 (30.41–30.78)	31.17 (31.63–30.7)	33.56 (33.34–33.78)	<0.001	ab: <0.001 ac: <0.001 ad: <0.001 bc: 0.049 bd: <0.001 cd: <0.001	31.83^*^ (31.69–31.99)	29.31^*^ (29.25–29.39)	33.02^*^ (32.82–33.23)	29.44^*^ (29.38–29.51)
	Adjusted	29.26 (29.2–29.3)	30.58 (30.44–30.72)	31.81(31.49–32.12)	33.38 (33.22–33.55)	<0.001	ab: <0.001 ac: <0.001 ad: <0.001 bc: <0.001 bd: <0.001 cd: <0.001	31.77^*^ (31.65–31.89)	29.35^*^ (29.29–29.41)	33.07^*^ (32.91–33.23)	29.45^*^ (29.39–29.5)
Hip circumference (cm)	Crude	76.79(76.54–77.04)	82.07 (81.38–82.75)	82.17 (0.01–84.32)	95.05 (94.24–95.85)	<0.001	ab: <0.001 ac: <0.001 ad: <0.001 bc: 0.999 bd: <0.001 cd: <0.001	87.5^*^ (86.93–88.07)	76.96^*^ (76.71–77.21)	92.13^*^ (91.3–92.96)	77.51^*^ (77.28–77.75)
	Adjusted	76.76(76.56–76.96)	82 (81.5–82.5)	84.7 (83.6–85.8)	96.65 (94–95.2)	<0.001	ab: <0.001 ac: <0.001 ad: <0.001 bc: <0.001 bd: <0.001 cd: <0.001	87.27^*^ (86.85-87.7)	77.1^*^ (76.89–77.32)	92.39^*^ (91.83–92.96)	77.56^*^ (77.36–77.76)
Wrist circumference(cm)	Crude	14.41 (14.38–14.45)	15.06 (14.97–15.15)	15.7 (15.5–15.91)	16.61 (16.51–16.71)	<0.001	ab: <0.001 ac: <0.001 ad: <0.001 bc: <0.001 bd: <0.001 cd: <0.001	15.72^*^ (15.65–15.79)	14.45^*^ (14.42–14.49)	16.41^*^ (16.32–16.5)	14.5^*^ (14.47–14.53)
	Adjusted	14.4 (14.38–14.44)	15.06 (14.99–15.13)	16 (15.85–16.15)	16.53 (16.45–16.61)	<0.001	ab: <0.001 ac: <0.001 ad: <0.001 bc: <0.001 bd: <0.001 cd: <0.001	15.69^*^ (15.63–15.74)	14.46^*^ (14.43–14.49)	16.43^*^ (16.36–16.51)	14.5^*^ (14.47–14.53)

a *Normal: 5th < BMI < 85th percentile and WHtR < 0.5*.

b *Only abdominal obesity: WHtR > 0.5 and BMI < 95th percentile*.

c *Only general obesity: BMI > 95th and WHtR < 0.5*.

d *Combined obesity: BMI > 95th and WHtR > 0.5*.

e *Abdominal obesity: WHtR > 0.5*.

f *General obesity: BMI > 95th*.

* *P ≤ 0.05 is considered as significant*.

*Adjusted for age, sex, PA, ST, SES and living area*.

Table 3 presents the mean values (95% CI) of HC, NC, and WrC according to metabolic status. Accordingly, a significant different association was reported between NC, HC, and WrC and metabolic phenotypes in the entire population. The average values of NC, HC, and WrC were significantly higher among students with the MNHO phenotype. Comparing the MHO and MNHNO phenotypes, the means of HC, NC, and WrC were higher in the MHO group. The highest means of NC, HC, and WrC were observed in the MNHO and MHO phenotypes, while the lowest means of NC, HC, and WrC were observed in the MHNO phenotype.

**Table 3:** Means (95%) of hip, neck, and wrist circumference according to metabolic status: the CASPIAN-V study.

	**Different combinations of metabolic phenotype**					***P*-values between groups**	***P*-values within groups (*post-hoc*)**
		**MHNO^**a**^ (*n* = 3,202)**	**MHO^**b**^ (*n* = 340)**	**MNHNO^**c**^ (*n* = 120)**	**MNHO^**d**^ (*n* = 68)**		
Neck circumference (cm)^*^	Crude	29.57 (29.44–29.7)	32.63 (32.14–33.12)	30.26 (29.63–30.88)	34.1 (33.16–35.04)	<0.001	ab: <0.001 ac: 0.205 ad: <0.001 bc: <0.001 bd: 0.019 cd: <0.001
	Adjusted	29.55 (29.44–29.66)	32.79 (32.46–33.12)	30.24 (29.69–30.79)	34.14 (33.41–34.87)	<0.001	ab: <0.001 ac: 0.016 ad: <0.001 bc: <0.001 bd: 0.001 cd: <0.001
Hip circumference (cm)^*^	Crude	77.91 (77.46–78.37)	90.8 (88.75–92.86)	81 (78.6–83.4)	96.35 (92.93–99.78)	<0.001	ab: <0.001 ac: 0.001 ad: <0.001 bc: <0.001 bd: 0.011 cd: <0.001
	Adjusted	77.81 (77.43–78.19)	91.66 (90.48–92.84)	81 (79.03–82.97)	96.83 (94.2–99.45)	<0.001	ab: <0.001 ac: 0.002 ad: <0.001 bc: <0.001 bd: <0.001 cd: <0.001
Wrist circumference (cm)^*^	Crude	14.55 (14.49–14.6)	16.28 (16.08–16.47)	15.14 (14.83–15.46)	16.99 (16.53–17.45)	<0.001	ab: <0.001 ac: 0.077 ad: <0.001 bc: <0.001 bd: 0.001 cd: <0.001
	Adjusted	14.54 (14.49–14.59)	16.35 (16.2–16.5)	15.14 (14.89–15.4)	16.98 (16.65–17.32)	<0.001	ab: <0.001 ac: <0.001 ad: <0.001 bc: <0.001 bd: 0.001 cd: <0.001

a *MHNO: Metabolically Healthy Non-Obese (healthy)*.

b *MHO: Metabolically Healthy Obese*.

c *MNHNO: Metabolically Non-Healthy Non-Obese*.

d *MNHO: Metabolically Non-Healthy Obese*.

* *P ≤ 0.05 is considered as significant*.

*Adjusted for age, sex, PA, ST, SES, and living area*.

Tables 4, 5 show the odds ratio in crude modeling and models adjusted for age, sex, physical activity, screening time, socioeconomic status, and living area. The association of HC, NC, and WrC with abdominal and general obesity in multinominal logistic regression models is presented in Table 4. The association of abdominal, general, and combined obesity with NC, WrC, and HC was statistically significant. According to Table 4, per one unit increment in NC, HC, and WrC, the odds of only abdominal obesity increased 17% (OR:1.17, CI: 1.15–1.2), 6% (OR:1.06, CI: 1.05–1.07), and 41% (OR:1.41,CI:1.36–1.47), respectively, compared to the normal subjects (adjusted for age, sex, PA, ST, SES, and living area). Also, per one unit increment in NC and HC, the odds of combined obesity increased 58% (OR: 1.58, CI: 1.54–1.62) and 24% (OR:1.24, CI: 1.23–1.25) compared to the normal subjects. Furthermore, in subjects with only general obesity, HC, NC, and WrC were 37% (OR: 1.37, 95% CI: 1.31–1.42), 10% (OR: 1.1, 95% CI: 1.08–1.12), and 2.14 times (OR: 2.14, 95% CI: 1.99–2.3) higher than in subjects who were normal, respectively (adjusted for age, sex, PA, ST, SES, and living area).

**Table 4:** Association of neck, hip, and wrist circumference with general, abdominal, and combined obesity in a multinomial logistic regression model.

			**Normal^**a**^**	**Only abdominal obesity^**b**^**	**Only general obesity^**c**^**	**Combined obesity^**d**^**
Neck circumference (cm)	Model I^e^	OR (95% CI)	Reference	1.1 (1.08–1.11)^*^	1.14 (1.11–1.17)^*^	1.32 (1.3–1.34)^*^
	Model II^f^	OR (95% CI)		1.17 (1.15–1.2)^*^	1.37 (1.31–1.42)^*^	1.58 (1.54–1.62)^*^
Hip circumference (cm)	Model I^e^	OR (95% CI)	Reference	1.03 (1.02–1.03)^*^	1.03 (1.02–1.04)^*^	1.11 (1.1–1.12)^*^
	Model II^f^	OR (95% CI)		1.06 (1.05–1.07)^*^	1.1 (1.08–1.12)^*^	1.24 (1.23–1.25)^*^
Wrist circumference (cm)	Model I^e^	OR (95% CI)	Reference	1.23 (1.2–1.27)^*^	1.48 (1.4–1.57)^*^	1.85 (1.78–1.91)^*^
	Model II^f^	OR (95% CI)		1.41 (1.36–1.47)^*^	2.14 (1.99–2.3)^*^	2.5 (2.38–2.62)^*^

a *Normal: 5th < BMI < 85th percentile and WHtR < 0.5*.

b *Only abdominal obesity: WHtR > 0.5 and BMI < 95th percentile*.

c *Only general obesity: BMI > 95th and WHtR < 0.5*.

d *Combined obesity: BMI > 95th and WHtR > 0.5*.

e *Crude model*.

f *Adjusted for age, sex, PA, ST, SES, and living area*.

* *p ≤ 0.05 is considered as significant*.

**Table 5:** Association of neck, hip, and wrist circumference with metabolic status in a multinomial logistic regression model.

	**Different obesity phenotypes**					
	**MHNO^**a**^**	**MHO^**b**^**	**MNHNO^**c**^**	**MNHO^**d**^**		
Neck Circumference (cm)	Model I^e^	OR (95% CI)	Reference	1.23 (1.19–1.27)^*^	1.05 (1–1.1)^*^	1.34 (1.26–1.43)^*^
	Model II^f^	OR (95% CI)		1.47 (1.4–1.54)^*^	1.08 (1.01–1.15)^*^	1.64 (1.51–1.79)^*^
Hip circumference (cm)	Model I^e^	OR (95% CI)	Reference	1.07 (1.06–1.08)^*^	1.01 (1.00–1.03)^*^	1.11 (1.09–1.13)^*^
	Model II^f^	OR (95% CI)		1.17 (1.15–1.19)^*^	1.03 (1.01–1.05)^*^	1.24 (1.2–1.28)^*^
Wrist circumference (cm)	Model I^e^	OR (95% CI)	Reference	1.73 (1.61–1.85)^*^	1.22 (1.1–1.35)^*^	2.11 (1.84–2.42)^*^
	Model II^f^	OR (95% CI)		2.38 (2.17–2.62)^*^	1.34 (1.17–1.54)^*^	2.96 (2.5–3.5)^*^

a *MHNO: Metabolically Healthy Non-Obese*.

b *MHO: Metabolically Healthy Obese*.

c *MNHNO: Metabolically Non-Healthy Non-Obese*.

d *MNHO: Metabolically Non-Healthy Obese*.

e *Crude model*.

f *Adjusted for age, sex, PA, ST, SES, and living area*.

* *p ≤ 0.05 is considered as significant*.

The association of the different metabolic phenotypes with the study anthropometric indices became significantly greater with increased NC, WrC, and HC compared to the healthy group (MHNO). The strongest association was seen when comparing the MNHO group with the MHNO group. According to Table 5, per one unit increment in NC and HC, the odds of MHO increased 47% (OR: 1.47, CI: 1.4–1.54) and 17% (OR: 1.17, CI: 1.15–1.19) compared to MHNO, respectively, and also, per one unit increase in WrC, the odds of MHO was 2.38 times greater than in the MHNO group (adjusted for age, sex, PA, ST, SES, and living area).

## Discussion

The findings of this study indicate that there are significant but different associations between the means of NC, HC, and WrC and the phenotypes of obesity and their metabolic status. Therefore, based on the collection of evidence from this relatively large sample, it can be stated that NC, HC, and WrC are probably predictors of obesity among the population.

Recently, NC, HC, and WrC have been reported as indicators of body fat distribution and to be simple and practical indices (36). Very limited studies are available that assess the association among NC, HC, WrC, and obesity phenoype or provide data for evaluating the potential of these indicators for obesity in the pediatric population (37–39).

The CASPIAN-IV Study shows that overweight students and those with general obesity and abdominal obesity had a relatively larger NC measurement than other students (37). The present study assessed individual groups of students with general as well as abdominal adiposity or both phenotypes. The results of our study demonstrated that students with both types of obesity had, on average, larger NC, HC, and WrC values than any of the other three types (normal, abdominally obese, and generally obese). Also, a comparison between the two types of obesity showed that students with general obesity had higher values for these indicators than those with abdominal obesity. Other studies have reported similar results, for example, the Framingham Heart Study (40) and the Turkish Adult Cohort Study (1). Considering the existence of a strong association between the WrC and other anthropometric measurements and also its association with both general obesity and abdominal obesity, it is possible to consider WrC as important as NC when predicting general and abdominal obesity (37).

Many studies conducted on the causes of the prevalence of obesity and metabolic syndrome disorder have indicated that the possibility of obesity in adulthood is higher among children who are not obese but suffer from metabolic syndrome disorder (41). These findings indicate the significance of protecting children and adolescents who are not obese but have metabolic syndrome disorders (MNHNO) against chronic metabolism-related diseases and also obesity, which will eventually lead to death in adulthood. Another conclusion derived from this study is that, regarding the higher NC, HC, and WrC values among non-obese students who suffer from metabolic syndrome compared MHO and also the higher values of these indicators among obese individuals with metabolic syndrome (MNHO) compared to other phenotypes, NC, HC, and WrC are probably strongly correlated with metabolic syndrome disorders. Also, another study has shown that individuals with metabolic syndrome have higher WrC than normal individuals (42). On the other hand, since WrC has a strong association with NC, weight, BMI, and body fat percentage, it can be generally concluded that people with higher NC are probably more at risk of being affected by metabolic syndrome disorder. There is a significant association between the results of this study and those of others conducted on children and adolescents, suggesting that the neck measurement is an acceptable predictor of higher BMI, obesity, and its related diseases (42, 43). Therefore, it can be said that NC, HC, and WrC are a low-cost, accessible, but valuable way of diagnosing obesity without metabolic syndrome (MHO) and metabolic syndrome without obesity (MNHNO). This finding can contribute to the prevention of serious diseases (e.g., cardiovascular diseases), decreasing the cost load on the health sector and reducing death rates.

Today, it is not possible to justify the increasing prevalence of obesity in adulthood only by genetic or lifestyle factors. Studies conducted in this area have indicated a combination of factors in childhood that affect obesity (12). The ease of taking measurements that can be predictive of obesity and its various phenotypes will be very helpful for recognizing children at high risk. The results of this study show that, considering normal students as a reference, students with comparatively NC and HC values are more susceptible to general obesity. Moreover, students with relatively high NC, HC, and WrC are more susceptible to obesity with metabolic syndrome (MNHO).

Due to its ease of measurement and interpretation, BMI is an indicator that is commonly used to evaluate weight status among children and adults; however, this method has certain limitations (44–46). WrC is a good indicator of the distribution of fat, skeletal structure, and also metabolic status in the body. Based on our findings, WrC is a better indicator of obesity phenotype and other metabolic and cardiovascular disorders among children and adolescents than BMI (43, 47, 48).

Based on the results of Capizzi et al., pubertal status could influence NC and WrC measurements as well as the metabolic parameters and bone component in overweight and obese children and adolescents (49); unfortunately, the pubertal status of the participants was not evaluated in the present study, which is one of its limitations. However, puberty and age are highly correlated with one another and can represent a reasonable proxy measure of maturity. Therefore, new measurements such as NC and WrC can be of great help in recognizing risk factors of cardiovascular diseases such as different types of general and abdominal obesity, metabolic syndrome disorders, and BMI during childhood or adolescence.

One of the strengths of this study, which is derived from the CASPIAN-V study, is its novelty in terms of including children and adolescents. No study has been conducted so far on the association of NC, HC, and WrC with different obesity phenotypes and their metabolic status in the population of Iranian students. However, the study had some limitations. Due to its cross-sectional nature, the causality could not be construed. Additionally, we were not able to measure fat mass.

## Conclusion

The current study demonstrated that in children and adolescents, HC, NC, and WrC were significantly but differentially associated with obesity phenotypes and their metabolic status. These indicators are innovative, low-cost, and alternative tools for assessing obesity and metabolic syndrome in different age and sex pediatric populations.

## Data Availability Statement

The datasets generated for this study are available on request to the corresponding author.

## Ethics Statement

After complete explanation of the study objectives and methods, written informed consent and verbal consent were obtained from the parents and students, respectively. The study protocol was reviewed and approved by ethical committees and other relevant national regulatory organizations. The Research and Ethics Council of Isfahan University of Medical Sciences approved the study (Project Number: 194049).

## Author Contributions

MP participated in the sequence alignment and drafted the manuscript. RK participated in the study design, final revision, and editing. NS participated in the sequence alignment and drafted the manuscript. MM and SH-R participated in the study design and interpretation. HZ, MAP, and GS participated in the data acquisition. MQ and RH participated in the design of the study and performed statistical analysis. All authors read and approved the final manuscript.

### Conflict of Interest

The authors declare that the research was conducted in the absence of any commercial or financial relationships that could be construed as a potential conflict of interest.
